# Respiratory physiological changes post initiation of neurally adjusted ventilatory assist in preterm infants with evolving or established bronchopulmonary dysplasia

**DOI:** 10.1007/s00431-025-05997-x

**Published:** 2025-01-29

**Authors:** Basma Mohamed, Anay Kulkarni, Donovan Duffy, Anne Greenough, Sandeep Shetty

**Affiliations:** 1 Neonatal Intensive Care Centre, St George’s University Hospitals NHS Foundation Trust, London, SW17 0QT UK; 2 George’s University of London, London, SW17 0QT UK; 3 Department of Women and Children’s Health, School of Life Course Sciences, Faculty of Life Sciences and Medicine, King’s College London, London, UK

**Keywords:** Neurally adjusted ventilatory assist, NAVA, Prematurity, Bronchopulmonary dysplasia, BPD

## Abstract

To assess respiratory changes after neurally adjusted ventilatory assist (NAVA) initiation in preterm infants with evolving or established bronchopulmonary dysplasia (BPD). Premature infants born less than 32 weeks gestation with evolving or established BPD initiated on invasive or non-invasive (NIV) NAVA were included. Respiratory data: PCO_₂_ and SpO₂/FiO₂ (S/F) ratio before and at 4, 24, 48 h post-NAVA initiation were collected. Eighty-eight infants, median GA 25.1 (range 22.7–30.3) weeks, with 191 NAVA episodes were included. Infants born < 32 weeks with evolving and established BPD showed improvements in PCO_₂_ and S/F ratio 48 h post-NAVA compared to prior: 7.6 (4.5–11.8) versus 8.1 (4.7–13.1) kPa; *p* < 0.001 and 285 (118–471) versus 276 (103–471); *p* = 0.013, respectively. Improvements were observed in invasive NAVA: 7.6 (4.5–11.8) versus 8.5 (4.7–12.4) kPa; *p* = 0.001, 290 (148–471) versus 271 (103–467); *p* = 0.002, and NIV-NAVA: 7.5 (4.6–11.7) versus 7.9 (5.2–13.1) kPa; *p* = 0.001, 283 (128–471) versus 294 (114–471); *p* = 0.002. Severe BPD infants had reductions in PCO_₂_ 48 h post-initiation: 7.2 (5.6–9.7) versus 8.0 (5.4–11.7) kPa; *p* = 0.002, with lower FiO₂ requirements 0.37 (0.21–0.65) versus 0.43 (0.21–0.8); *p* = 0.011, and improved S/F ratios 263 (146–471) versus 219 (114–457); *p* = 0.006. On subgroup analysis, similar improvements were noted in; PCO_2_ levels in invasive NAVA (*p* = 0.011) and NIV-NAVA (*p* = 0.002), S/F ratios in invasive NAVA (*p* = 0.046) and NIV-NAVA (*p* = 0.002) and FiO₂ in invasive NAVA (*p* = 0.034) and NIV-NAVA (*p* = 0.053).

*Conclusion*: NAVA improves CO_₂_ clearance and oxygenation in infants with evolving or established and severe BPD at 48 h post-initiation. In severe BPD, NAVA also reduced oxygen requirements

**Table:** 

**What is Known:** • *NAVA has the potential to improve CO*_*2*_ *clearance and oxygenation by optimising alveolar ventilation, adapting to the infant's breathing patterns, and enhancing gas exchange.*	
**What is New:** • *The beneficial effects of NAVA are sustained in infants with evolving or established bronchopulmonary dysplasia (BPD), improving carbon dioxide clearance and oxygenation at 48 hours after initiation.*

**Supplementary Information:**

The online version contains supplementary material available at 10.1007/s00431-025-05997-x.

## Introduction

Despite advances in neonatal care that have improved survival rates for extremely preterm infants, the incidence of bronchopulmonary dysplasia (BPD) has remained largely unchanged over the past two decades [1]. BPD primarily results from mechanical ventilation and oxygen exposure in vulnerable infants. This condition can lead to long-term respiratory complications and neurodevelopmental abnormalities [2, 3].

In recent years, innovative ventilation strategies such as Neurally Adjusted Ventilatory Assist (NAVA) have emerged as potential solutions to improve respiratory support in BPD infants [4, 5]. NAVA synchronises ventilatory assistance using the infant’s neural respiratory drive, which may enhance comfort and reduce the need for sedation while optimising gas exchange [6]. NAVA utilises the electrical activity of the diaphragm to trigger the ventilator. A modified nasogastric feeding tube with a series of electrodes allows monitoring of the diaphragmatic electromyogram (Edi). The waveform of the Edi is used to trigger and control ventilator support.

NAVA has the potential to enhance CO_2_ clearance by optimising alveolar ventilation, adapting to the infant’s breathing patterns, and improving gas exchange [7]. This, because the breaths are proportionally synchronised both in timing and magnitude and the baby is more settled, CO_2_ clearance is better. In a randomised crossover trial [8], twenty-four patients were ventilated with NAVA or synchronised intermittent mandatory ventilation with pressure controlled and supported breath for 12 h and then crossed over to the alternative mode for 12 h. The median (range) GA at birth was 26.3 (23 to 39) weeks and at the time of study enrolment had a median (range) corrected gestational age (cGA) of 32.1 (28.7 to 44.6) weeks. The median age at study enrolment was 40 days (3 to 135 days). NAVA was associated with reduced PIPs, increased RR and higher PCO_2_ levels while maintaining an equivalent oxygen requirement. Although respiratory rates were higher, this was offset by lower peak inspiratory pressures during NAVA. These are encouraging results, but the infants were only studied up to 12 h on each mode.

Our previous study [9], determined if very prematurely born infants with evolving or established BPD had a lower oxygenation index (OI) on NAVA compared to assist control ventilation (ACV). Infants were studied for one hour on each mode. Nine infants, median GA of 25 (range 22–27) weeks, were studied at a median postnatal age of 20 (range 8–84) days. The mean OI after one hour on NAVA was 7.9 compared to 11.1 on ACV (*p* = 0.0007). The FiO_2_ (0.36 versus 0.45, *p* = 0.007), PIP (16.7 versus 20.1 cm H_2_O, *p* = 0.017) and MAP (9.2 versus 10.5 cm H_2_O, *p* = 0.004) were lower on NAVA. Despite encouraging results, the infants were only studied one hour on each mode.

The ratio of arterial oxygen partial pressure and fraction of inspired oxygen (PaO_2_/FiO_2_; P/F) although a good outcome predictor, requires arterial blood gas sampling which is invasive and not readily available in clinical practice, especially for children [10]. The oxygen saturation (SpO_2_)/FiO_2_ (S/F) ratio is a non-invasive, easily detectable, and readily available parameter that may be used as a surrogate marker for P/F in infants and children [10–12].

The aim of our study, therefore, was to determine whether NAVA had beneficial effects on key respiratory parameters such as the S/F ratio especially up to 48 h post-initiation.

## Methods

A retrospective study was undertaken. Premature infants born less than 32 weeks of gestational age with evolving or established BPD were started on NAVA at the discretion of the attending neonatal consultant who used local guidance on the use of NAVA. All infants born less than 32 weeks of gestation who were commenced on invasive or non-invasive NAVA between June 2019 and January 2024 were included in the study. This project was registered with St. George’s University Hospitals NHS Foundation Trust (SGH) clinical effectiveness department (reference number AUDI03552).

Premature infants born less than 32 weeks of gestational age requiring invasive ventilation support beyond the second week (14 days) of postnatal age were classified as infants with evolving BPD and premature infants born less than 32 weeks of gestational age requiring respiratory support at 36 weeks cGA were deemed infants who had established BPD. BPD was defined as need of oxygen at corrected GA 36 weeks as per NICHD definition [3, 13]. Infants needing NIV-PC (Presssure Control), BiPAP (bi-level positive airway pressure) at cGA of age were classified as having severe BPD. Infants were identified from a standardised electronic neonatal database (Badgernet) and our department’s own database for patients on NAVA. Data were obtained from the electronic documentation recording system and the medical notes.

Conventional invasive modes of ventilation prior to initiation of NAVA/NIV-NAVA included flow triggered pressure regulated volume-controlled ventilation (PRVC), Pressure Control ventilation (PC), synchronised intermittent mandatory ventilation (SIMV) with Pressure Support (PS) and high-frequency oscillatory ventilation (HFOV), and conventional non-invasive modes were such NIV-PC (NIV-Pressure control), CPAP (continuous positive airway pressure) and high-flow nasal cannula oxygen (HHFNC). Background mandatory respiratory rates (RR) were set between 30 and 40 breaths/min to allow spontaneous triggered inflation. Set pressure when on NIV-PC varied between 15–40/5–8 cm of H_2_O with backup RR between 30 and 40/min. Masks and bi-nasal prongs for NIV modes of ventilation were from Henleys medical supply. The same interface, bonnets, CPAP generator, ventilation circuit and ventilator were used before and after initiation of NAVA/NIV-NAVA.

NAVA/NIV-NAVA was delivered by the SERVO-n® Maquet Getinge ventilator. When on NAVA, a six French 49 or eight French 50 cm Edi catheter was inserted via the oro-gastric or nasogastric route and correct positioning confirmed as per the instructions of the manufacturer using the Edi catheter positioning guide function on the ventilator (Maquet Servo-n User Manual Version 4.1). The apnoea time was set to two seconds, and the upper pressure limit is at least 5 cm H_2_O higher than the baseline settings.

Outcome parameters were PCO_2_, peak inspiratory pressure (PIP), NAVA level, respiratory rate (RR) and oxygen requirement (FiO_2_) and SpO_2_/FiO_2_ (S/F) ratio were collected. These parameters were measured 4, 24, 48 h before and 4, 24, and 48 h after the initiation of NAVA except PIP and RR results which were collected only at 4 h.

### Statistical analysis

Differences in respiratory parameters, RR, MAP, S/F ratio, FiO_2_ requirement and PCO_2_ before and after NAVA/NIV-NAVA at each timepoint were assessed for statistical significance using the Wilcoxon signed rank test as appropriate using IBM SPPS statistical software, V.29 (IBM Corporation, USA).

## Results

Eighty-eight (54% male) infants were included in the study, who had a median GA of 25.1 weeks (range 22.7–30.3 weeks) and a median birth weight (BW) of 690 g (390–1260 g) (Table 1). The median (range) cGA when NAVA was initiated was 29.6 weeks (24.3–49 weeks) at a median postnatal age of 27 days (14–170 days). Antenatal corticosteroids were administered to 83 infants (94.3%), and postnatal steroids to 52 infants (87.6%). All infants received postnatal caffeine. During the hospital admission, 59 infants (67%) received NAVA on more than one occasion, resulting in a total of 191 NAVA episodes. Of these, 90 episodes (47.1%) were invasive NAVA.

**Table 1:** Demographic data. Data demonstrated as median (range)/percentage (%)

Gestational age (weeks)	25.1 (22.7–30.3)
Corrected gestational age (weeks at NAVA initiation)	29.6 (24.3–49)
Post-natal age (days)	27 (14–170)
Birth weight (g)	680 (390–1260)
Gender M/total (%)	48/88 (54%)
Chorioamnionitis/total (%)	8/88 (9.1%)
Mode of delivery: SVD/total (%)	59/88 (67%)
APGAR score at 5 min	6 (1–10)
Antenatal corticosteroid	83/88 (94.3%)
CRIB-II Score	12 (6–16)

Prior to initiation of NAVA/NIV-NAVA, invasive modes were PRVC in 38/90 (42%), PC in 51/90 (57%), HFOV (1%) in 1/90 episodes, non-invasive modes were NIV-PC in 63/101 (62%), CPAP in 30/101 (30%) and HHFNC in 8/101 (8%) episodes were noted. Spontaneous RR/min were 30–40 in 11/191 (6%), 40–50 in 77/191 (40%), 50–60 in 78/191 (41%) and 60–70 in 25/191 (13%) episodes. Positive end expiratory pressure (PEEP) set were between 5 and 8 cm of H_2_o, PIPs set or generated were between 15 and 40 cm of H_2_O, MAP when on HFOV was 10 cm of H_2_O and flow on HHFNC was 8 L/min. Three infants were on weaning doses of sedation of oral clonidine and morphine. Of these, two infants were on clonidine doses between 1 and 3 µg/kg/day and one infant was on oral morphine at 20 µg/kg/day.

**Fig. 1:**
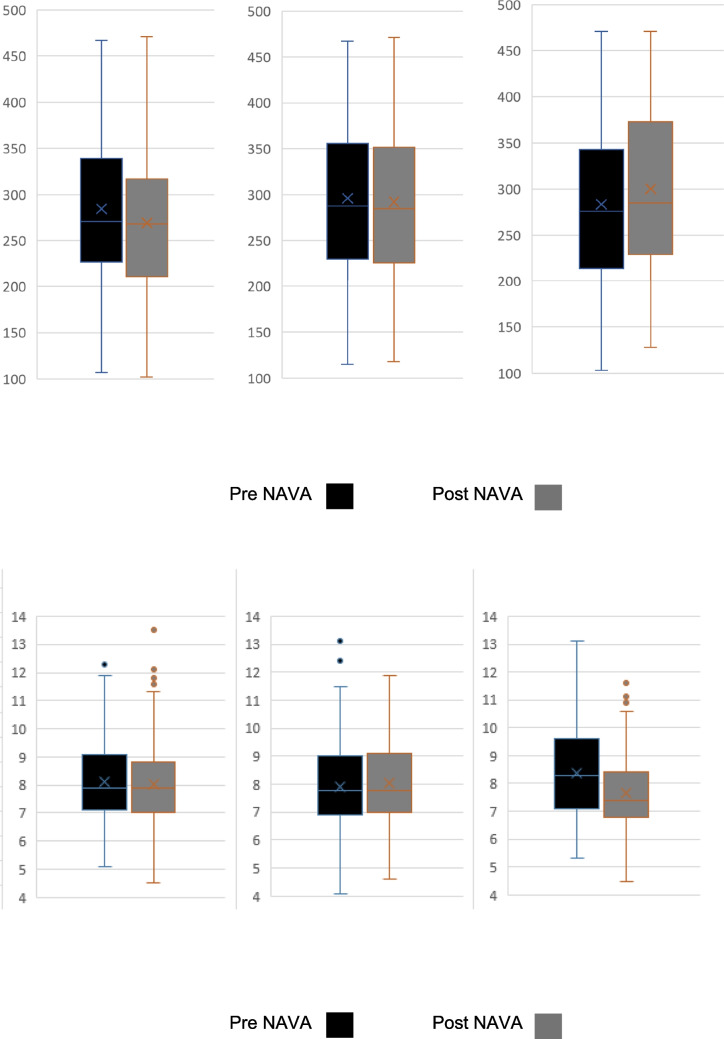
(**A**, **B**) SF ratio and PCO_2_ values for preterm infants with evolving or established BPD

**Fig. 2:**
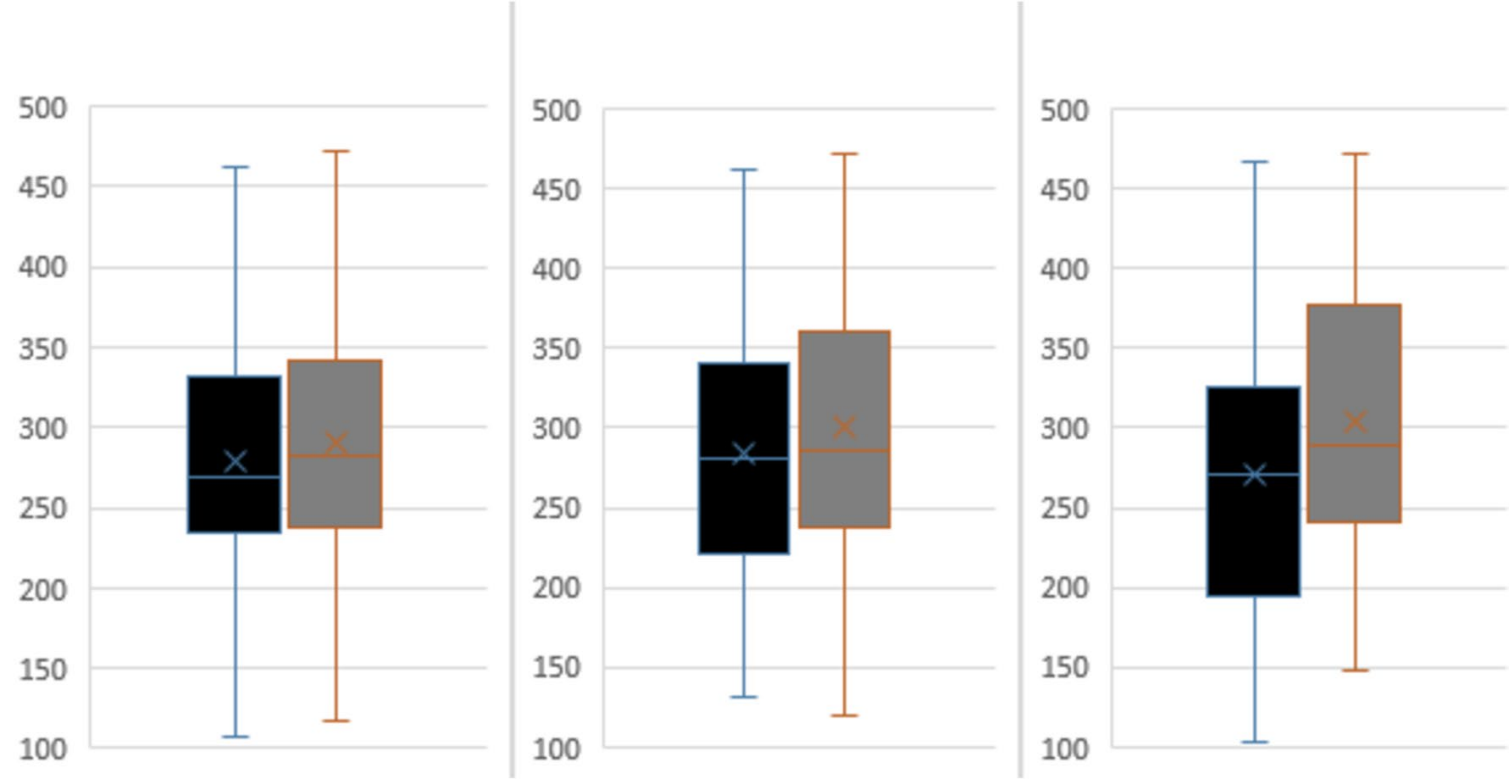
(**A**, **B**) SF ratio and PCO_2_ values for preterm infants with evolving or established BPD who required invasive NAVA

**Fig. 3:**
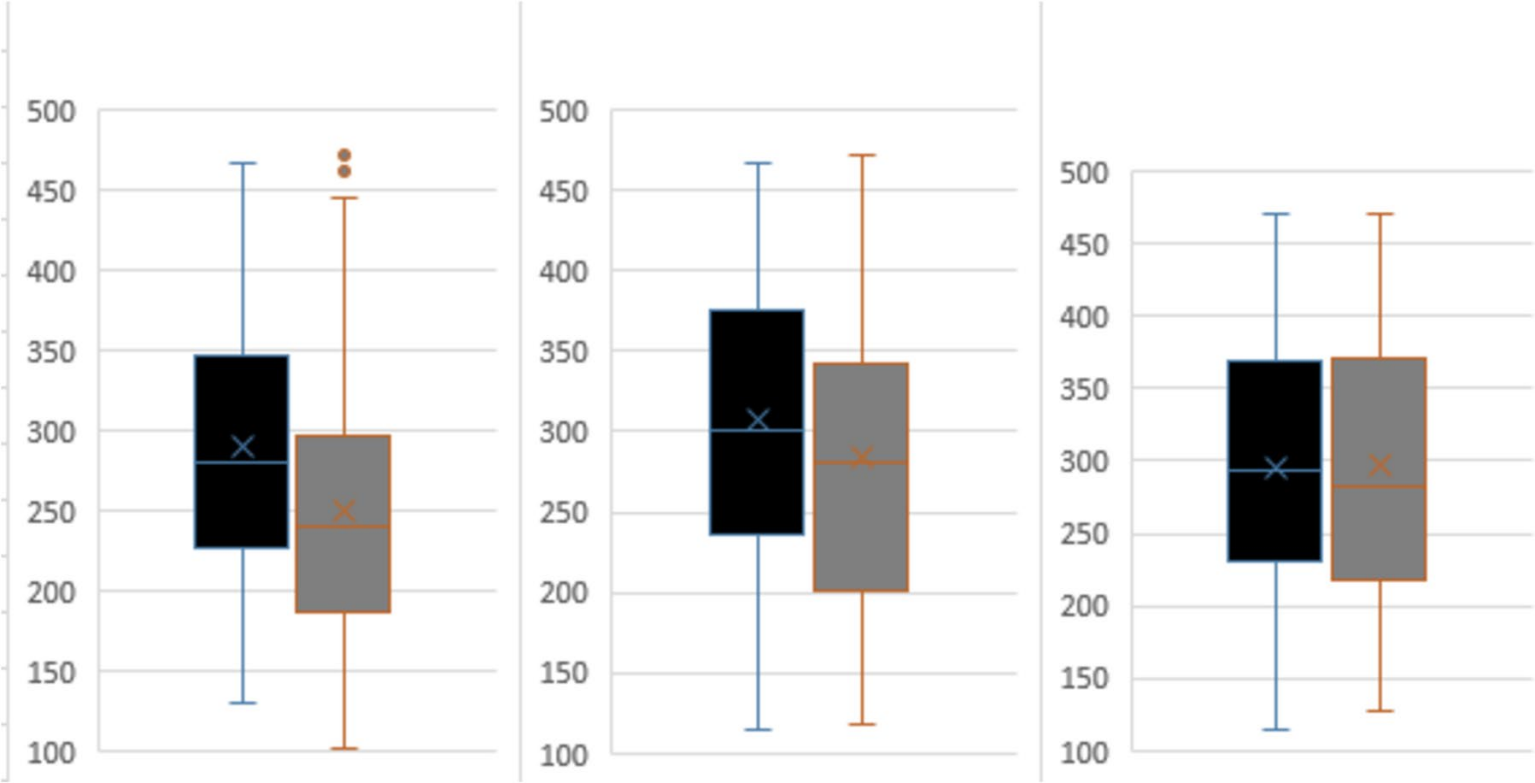
(**A**, **B**) S/F ratio and PCO_2_ values for preterm infants with evolving or established BPD who required non-invasive NAVA

Infants born less than 32 weeks gestation with evolving and established BPD and initiated on NAVA demonstrated significant improvements in PCO_2_ levels and S/F ratios 48 h post-NAVA initiation compared to prior: 7.6 (4.5–11.8) versus 8.1 (4.7–13.1) kPa; *p* < 0.001 and 285 (118–471) versus 276 (103–471); *p* = 0.013 respectively. (Fig. 1, Supplementary Table 1). There was no significant difference in PIP generated 21 (9–31) versus 20 (9–35); *p* = 0.412 and spontaneous RR at 4 h 53 (32–75) versus 55 (33–78); *p* = 0.506 post initiation. On subgroup analysis, this improvement in PCO_2_ and S/F ratio was in both infants on invasive NAVA:7.6 (4.5–11.8) versus 8.5 (4.7–12.4) kPa; *p* = 0.001, 290 (148–471) versus 271 (103–467); *p* = 0.002, (Fig. 2, Supplementary Table 2) and for those on NIV-NAVA: 7.5 (4.6–11.7) versus 7.9 (5.2–13.1) kPa; *p* = 0.001, 283 (128–471) versus 294 (114–471); *p* = 0.002 (Fig. 3, Supplementary Table 3).

In infants with severe BPD (*n* = 30), invasive NAVA (*n* = 13) and NIV-NAVA (*n* = 17), in whom NAVA was initiated after 36 weeks cGA, significant reductions in PCO_2_ levels were observed 48 h post-initiation 7.2 (5.6–9.7) versus 8.0 (5.4–11.7) kPa; *p* = 0.002, and lower FiO_2_ requirements 0.37 (0.21–0.65) versus 0.43 (0.21–0.8); *p* = 0.011 and improved S/F ratio 263 (146–471) versus 219 (114–457); *p* = 0.006 (Supplementary Table 4). On sub-group analysis (Supplementary Tables 5 and 6), the improvements in the FiO_2_ requirement observed 48 h post initiation in both those on invasive NAVA 0.51 (0.21–0.63) versus 0.51 (0.21–0.8); *p* = 0.034 and NIV-NAVA 0.36 (0.22–0.65) versus 0.36 (0.24–0.8); *p* = 0.053 and in S/F ratio were observed in those on invasive NAVA 262 (154–471) versus 188 (132–457); *p* = 0.046 and NIV-NAVA 267 (146–445) versus 258 (114–400); *p* = 0.002. Similarly, PCO_2_ levels improved 48 h post-initiation in invasive NAVA 6.9 (5.6–9.7) versus 8.1 (6.4–11.3); *p* = 0.011 and NIV-NAVA 7.4 (5.8–8.8) versus 7.9 (5.4–11.7); *p* = 0.002 (Supplementary Tables 5 and 6).

## Discussion

We have demonstrated that in prematurely born infants with evolving or established BPD NAVA/NIV-NAVA improved oxygenation and PCO_2_ levels 48 h post NAVA initiation compared to values 48 h prior. This effect was seen in infants on both invasive and non-invasive NAVA modes of ventilation. In infants with severe BPD in whom NAVA was initiated post 36 weeks cGA there were reductions in FiO_2_ requirement in addition to improvements in oxygenation and CO_2_ levels. Our results demonstrate a longer beneficial effect of NAVA than has been previously reported [14, 15].

Our results are similar to a previously published prospective crossover study [14], but that study only included five ventilated infants born between GA of 25 and 29 weeks and only for four hours on PCV and fours on NAVA. However, in that study on NAVA the infants required less PIP and RR to achieve lower PCO_2_ which was not seen in our study. In a crossover study [15], in which 25 infants were given CPAP and NAVA there were no significant differences in partial pressure of carbon dioxide (PaCO_2_) between the two modes, and both were in normal range [15]. The study however took place in a paediatric intensive care unit and so dissimilar to the presently reported cohort of preterm infants with evolving or established BPD.

In a retrospective matched cohort study [7], 29 preterm infants with a median gestational age of 25.4 weeks (range, 23.4–30.3 weeks) who required over four weeks of mechanical ventilation and had a respiratory severity score (RSS) greater than four were assessed. Key respiratory parameters were compared before and after transitioning to NAVA at 1, 4, 12, and 24 h. The postmenstrual age at NAVA conversion ranged from 26.4 to 43.3 weeks. The median duration on conventional ventilation was 52.0 days, and on NAVA was 18.5 days. Significant improvements were noted in PCO₂, FiO₂, SpO₂, with reduced oxygen requirements and enhanced oxygen saturation at 4, 12, and 24 h post-transition [7].

Our study showed similar results in carbon dioxide clearance and oxygenation with improved FiO_2_ requirement in the severe BPD group but importantly beyond 24 h. However, no significant improvement was observed in MAP and PIP levels.

The strength of our study is that the assessment period was for 48 h before and after NAVA initiation with a large sample size. This period of assessment was longer than many reported studies. Moreover, the ventilator and interface used were the same before and after use of NAVA. Only three infants were on weaning doses of oral sedation which was the same dose before and after initiation of NAVA/NIV-NAVA, hence unlikely to have influenced the respiratory effort and consequently the CO_2_ clearance. We calculated the S/F ratio s which gives better information on the oxygenation response than just FiO_2_ alone [11, 12].

In conclusion, NAVA has beneficial effects for infants with evolving or established BPD in improving oxygenation and CO_2_ clearance at 48 h and in those with severe BPD in CO_2_ clearance, oxygenation, and oxygen requirement.

## Supplementary Information

Below is the link to the electronic supplementary material.

Supplementary file1 (DOCX 23 KB)

## Data Availability

No datasets were generated or analysed during the current study.

## Abbreviations

ACV: Assist Control ventilation
BiPAP: Bi-level positive airway pressure
BPD: Bronchopulmonary dysplasia
cGA: Corrected gestational age
CPAP: Continuous positive airway pressure
EAdi: Electrical activity of the diaphragm
FiO_2_: Fraction of inspired oxygen
GA: Gestational age
h: Hour
HFOV: High-frequency oscillatory ventilation
HHFNC: High-flow nasal cannula
IVH: Intraventricular haemorrhage
kPa: Kilopascal
MAP: Mean airway pressure
NAVA: Neurally adjusted ventilatory assist
NIV: Non-invasive ventilation
NIV-PC: NIV-Pressure control
OI: Oxygenation index
PaCO_2_: Partial arterial pressure of carbon dioxide
PC: Pressure Control
PCO_2_: Partial pressure of carbon dioxide
PEEP: Positive end expiratory pressure
PIP: Peak inspiratory pressure
PRVC: Pressure regulated volume-controlled ventilation
PS: Pressure Support
RR: Respiratory rate
RSS: Respiratory severity score
S/F ratio: SpO_2_/FiO_2_
SGH: St. George’s University Hospitals NHS Foundation Trust
SIMV: Synchronised intermittent mandatory ventilation
SpO2: Pulse oximetric saturation
