# Supplementary material for: Respiratory physiological changes post initiation of neurally adjusted ventilatory assist in preterm infants with evolving or established bronchopulmonary dysplasia
Source: Eur J Pediatr. 2025 Jan 29;184(2):159. doi: 10.1007/s00431-025-05997-x (PMC11779694; doi:10.1007/s00431-025-05997-x)
Supplement: Supplementary file 1 — Supplementary file1 (DOCX 23 KB) [file 431_2025_5997_MOESM1_ESM.docx]

**Supplement Table 1: Respiratory physiological parameters for preterm infants with evolving or established BPD. Data are presented as median (range)**

|  | **Pre NAVA (n= 191)** | **Post NAVA (n= 191)** | **P value** |
| --- | --- | --- | --- |
| FiO_2_ at 4h | 0.34 (0.21 - 0.85) | 0.35 (0.21 - 0.80) | <0.001 |
| FiO_2_ at 24h | 0.33 (0.21 - 0.7) | 0.35 (021 - 0.80) | 0.140 |
| FiO_2_ at 48h | 0.33 (0.21 - 0.8) | 0.34 (0.21 - 0.75) | 0.4 |
| PCO_2_ at 4h kPa | 7.9 (5.1 - 12.3) | 7.9 (4.5 - 13.5) | 0.266 |
| PCO_2_ at 24h kPa | 7.8 (4.1 - 13.1) | 7.8 (4.6 - 11.9) | 0.532 |
| PCO_2_ at 48h kPa | 8.1 (4.7 - 13.1) | 7.6 (4.5 - 11.8) | <0.001 |
| S/F ratio at 4h | 271 (107 - 467) | 268 (102 - 471) | 0.009 |
| S/F ratio at 24h | 288 (115 - 467) | 285 (118 - 471) | 0.664 |
| S/F ratio at 48h | 276 (103 - 471) | 285 (128 - 471) | 0.013 |
|  |  |  |  |
|  |  |  |  |
|  |  |  |  |

**Supplement Table 2: Respiratory physiological parameters for preterm infants with evolving or established BPD who required invasive NAVA. Data are presented as median (range)**

|  | **Pre NAVA (n= 90)** | | **Post NAVA (n= 90)** | **P value** |
| --- | --- | --- | --- | --- |
| FiO_2_ at 4h | | 0.34 (0.21 - 0.85) | 0.34 (0.21 - 0.8) | 0.168 |
| FiO_2_ at 24h | | 0.33 (0.21 - 0.7) | 0.35 (021 - 0.80) | 0.231 |
| FiO_2_ at 48h | | 0.34 (0.21 - 0.8) | 0.35 (0.21 - 0.75) | 0.053 |
| PCO_2_ at 4h kPa | | 8.2 (5.2 - 11.9) | 8.2 (4.5 - 12.1) | 0.08 |
| PCO_2_ at 24h kPa | | 8.2 (4.1 - 13.1) | 7.9 (4.6 - 11.9) | 0.541 |
| PCO_2_ at 48h kPa | | 8.5 (4.7 - 12.4) | 7.6 (4.5 - 11.8) | 0.001 |
| S/F ratio at 4h | | 269 (107 - 462) | 283 (118 - 471) | 0.07 |
| S/F ratio at 24h | | 280 (132 - 462) | 287 (119- 471) | 0.057 |
| S/F ratio at 48h | | 271 (103 - 467) | 290 (148 - 471) | 0.002 |
|  | |  |  |  |
|  | |  |  |  |
|  | |  |  |  |

**Supplement Table 3: Respiratory physiological parameters for preterm infants with evolving or established BPD who required non-invasive NAVA. Data are presented as median (range)**

|  | **Pre NAVA (n=101)** | **Post NAVA (n=101)** | **P value** |
| --- | --- | --- | --- |
| FiO_2_ at 4h | 0.34 (0.21 - 0.7) | 0.31 (0.21 - 0.8) | 0.168 |
| FiO_2_ at 24h | 0.31 (0.21 - 0.65) | 0.35 (021 - 0.8) | 0.231 |
| FiO_2_ at 48h | 0.32 (0.21- 0.8) | 0.35 (0.21 - 0.75) | 0.053 |
| PCO_2_ at 4h kPa | 7.7 (5.1 - 12.3) | 7.9 (4.7 - 13.5) | 0.08 |
| PCO_2_ at 24h kPa | 7.6 (4.9 - 11.3) | 7.7 (4.8 - 11.9) | 0.541 |
| PCO_2_ at 48h kPa | 7.9 (5.2 - 13.1) | 7.5 (4.6 - 11.7) | 0.001 |
| S/F ratio at 4h | 279 (130 - 467) | 240 (102 - 471) | 0.07 |
| S/F ratio at 24h | 300 (115 - 467) | 280 (118 – 471) | 0.057 |
| S/F ratio at 48h | 294 (114 - 471) | 283 (128 - 471) | 0.002 |

**Supplement Table 4: Respiratory physiological parameters for preterm infants with severe BPD Data are displayed as median (range)**

|  | **Pre NAVA (n= 30)** | **Post NAVA (n=30)** | **P value** |
| --- | --- | --- | --- |
| FiO_2_ at 4h | 0.37 (0.25 - 0.85) | 0.39 (0.22 - 0.7) | 0.839 |
| FiO_2_ at 24h | 0.39 (0.22 - 0.7) | 0.41 (0.21 - 0.8) | 0.715 |
| FiO_2_ at 48h | 0.43 (0.21 - 0.8) | 0.37 (0.21 - 0.65) | 0.011 |
| PCO_2_ at 4h kPa | 7.35 (5.9 - 10.5) | 7.6 (5 - 11.5) | 0.902 |
| PCO_2_ at 24h kPa | 7.6 (5 - 11.5) | 7.5 (5.4 - 10.9) | 0.965 |
| PCO_2_ at 48h kPa | 7.9 (5.4 - 11.7) | 7.2 (5.6 - 9.7) | 0.002 |
| SF ratio at 4h | 253 (107 - 388) | 228 (118 - 441) | 0.765 |
| SF ratio at 24h | 244 (115 - 445) | 236 (118 - 471) | 0.773 |
| SF ratio at 48h | 219 (114 - 457) | 263 (146 - 471) | 0.006 |
|  |  |  |  |
|  |  |  |  |
|  |  |  |  |

**Supplement Table 5: Respiratory physiological parameters for preterm infants with severe BPD who required invasive NAVA. Data are demonstrated as median (range)**

|  | **Pre NAVA (n= 13)** | **Post NAVA (n=13)** | **P value** |
| --- | --- | --- | --- |
| FiO_2_ at 4h | 0.4 (0.25 - 0.85) | 0.37 (0.22 - 0.8) | 0.033 |
| FiO_2_ at 24h | 0.4 (0.25 - 0.7) | 0.4 (0.21 - 0.8) | 0.576 |
| FiO_2_ at 48h | 0.51 (0.21 - 0.8) | 0.51 (0.21 - 0.63) | 0.034 |
| PCO_2_ at 4h kPa | 7.3 (6.1 - 10.5) | 7.6 (5.6 - 9.5) | 0.506 |
| PCO_2_ at 24h kPa | 7.8 (5 - 11.5) | 7.4 (5.4 - 10.9) | 0.367 |
| PCO_2_ at 48h kPa | 8.1 (6.4 -11.3) | 6.9 (5.6 - 9.7) | 0.011 |
| SF ratio at 4h | 243 (107 - 388) | 262 (118 - 441) | 0.152 |
| SF ratio at 24h | 238 (132 - 380) | 243 (119 - 467) | 0.507 |
| SF ratio at 48h | 188 (132 - 457) | 262 (154 - 471) | 0.046 |
|  |  |  |  |
|  |  |  |  |
|  |  |  |  |

**Supplement Table 6: Respiratory physiological parameters for preterm infants with severe BPD who required NIV-NAVA. Data are demonstrated as median (range)**

|  | **Pre NAVA (n= 17)** | **Post NAVA (n=17)** | **P Value** |
| --- | --- | --- | --- |
| FiO_2_ at 4h | 0.37 (0.26 - 0.65) | 0.43 (0.23 - 0.8) | 0.168 |
| FiO_2_ at 24h | 0.37 (0.22 - 0.6) | 0.42 (0.21 - 0.8) | 0.231 |
| FiO_2_ at 48h | 0.36 (0.24 - 0.8) | 0.36 (0.22 - 0.65) | 0.053 |
| PCO_2_ at 4h kPa | 7.4 (5.9 - 9.9) | 7.6 (5.9 - 9.9) | 0.08 |
| PCO_2_ at 24h kPa | 7.3 (5.3 - 11.3) | 7.5 (5.4 - 9.3) | 0.541 |
| PCO_2_ at 48h kPa | 7.9 (5.4 -11.7) | 7.4 (5.8 - 8.8) | 0.001 |
| SF ratio at 4h | 257 (140 - 369) | 214 (118 - 426) | 0.07 |
| SF ratio at 24h | 262 (115 – 445) | 229 (118 - 471) | 0.057 |
| SF ratio at 48h | 258 (114 - 400) | 267 (146 - 445) | 0.002 |
|  |  |  |  |
|  |  |  |  |
